# Radiation and Chemical Program Research for Multi-Utility and Repurposed Countermeasures: A US Department of Health and Human Services Agencies Perspective

**DOI:** 10.1017/dmp.2023.226

**Published:** 2024-02-22

**Authors:** Carmen I. Rios, Efrain E. Garcia, Thomas S. Hogdahl, Mary J. Homer, Narayan V. Iyer, Judith W. Laney, Shannon G. Loelius, Merriline M. Satyamitra, Andrea L. DiCarlo

**Affiliations:** 1 Radiation and Nuclear Countermeasures Program (RNCP), National Institute of Allergy and Infectious Diseases (NIAID), National Institutes of Health (NIH), US Department of Health and Human Services (HHS), Washington, DC, USA; 2 Chemical Medical Countermeasures (MCM) Program, Biomedical Advanced Research and Development Authority (BARDA), Administration for Strategic Preparedness and Response (ASPR), Washington, DC, USA; 3 Burn/Blast MCM Program, Biomedical Advanced Research and Development Authority (BARDA), Administration for Strategic Preparedness and Response (ASPR), Washington, DC, USA; 4 Radiological/Nuclear MCM Program, Division of Chemical, Biological, Radiological, and Nuclear Medical Countermeasures, Biomedical Advanced Research and Development Authority (BARDA), Administration for Strategic Preparedness and Response (ASPR), HHS, Washington, DC, USA

**Keywords:** Chemical and Radiological Threats, Sulfur Mustard, Skin and lung injury, Medical countermeasures

## Abstract

Although chemical and radiological agents cause toxicity through different mechanisms, the multiorgan injuries caused by these threats share similarities that convene on the level of basic biological responses. This publication will discuss these areas of convergence and explore “multi-utility” approaches that could be leveraged to address common injury mechanisms underlying actions of chemical and radiological agents in a threat-agnostic manner. In addition, we will provide an overview of the current state of radiological and chemical threat research, discuss the US Government’s efforts toward medical preparedness, and identify potential areas for collaboration geared toward enhancing preparedness and response against radiological and chemical threats. We also will discuss previous regulatory experience to provide insight on how to navigate regulatory paths for US Food and Drug Administration (FDA) approval/licensure/clearance for products addressing chemical or radiological/nuclear threats. This publication follows a 2022 trans-agency meeting titled, “Overlapping Science in Radiation and Sulfur Mustard Exposures of Skin and Lung: Consideration of Models, Mechanisms, Organ Systems, and Medical Countermeasures,” sponsored by the National Institute of Allergy and Infectious Diseases (NIAID), a part of the National Institutes of Health (NIH). Discussions from this meeting explored the overlapping nature of radiation and chemical injury and spurred increased interest in how preparedness for one threat leads to preparedness for the other. Herein, subject matter experts from the NIAID and the Biomedical Advanced Research and Development Authority (BARDA), a part of the Administration for Strategic Preparedness and Response (ASPR), summarize the knowledge gained from recently funded biomedical research, as well as insights from the 2022 meeting. These topics include identification of common areas for collaboration, potential use of biomarkers of injury to identify injuries caused by both hazards, and common and widely available treatments that could treat damage caused by radiological or chemical threats.

Over the last 20 years, extensive efforts have been undertaken across the US Government (USG), industry, and academia to better understand and prepare for new and emerging threats involving chemical, biological, radiological, or nuclear (CBRN) material. The terrorist attacks on September 11, 2001, led the USG to establish a civilian-focused, medical countermeasures (MCMs) development program directed by the Department of Health and Human Services (HHS). Within the HHS, the charge to conduct early-stage research and development of radiological/nuclear and chemical MCMs was assigned to the National Institute of Allergy and Infectious Diseases (NIAID), a division of the National Institutes of Health (NIH), under the aegis of the Radiation and Nuclear Countermeasures Program (RNCP) and the Chemical Countermeasures Research Program (CCRP). The Biomedical Advanced Research and Development Authority (BARDA) within the Administration for Strategic Preparedness and Response (ASPR) is charged with activities to develop MCMs that address the public health and medical consequences of CBRN incidents, pandemic influenza, and emerging infectious diseases. BARDA uses innovative technologies to enhance preparedness against potential known and unknown threats. ASPR collaborates with hospitals, health care coalitions, biotechnology firms, community members, state, local, tribal and territorial governments, and other partners across the country to improve readiness and response capabilities. Under BARDA, the Chemical MCM, Radiological and Nuclear MCM, and Burn and Blast MCM Programs partner with industry, academia, and other government agencies to develop treatments for injuries caused by exposure to chemical, radiological, and nuclear threats.

ASPR also delivers to and maintains assets in the Strategic National Stockpile (SNS), having assumed responsibility for the SNS from the Centers for Disease Control and Prevention (CDC) in 2018. The SNS is part of the federal medical response infrastructure and can supplement MCMs needed by states, tribal nations, territories, and the largest metropolitan areas during public health emergencies.

This paper describes a trans-agency perspective on the strategies that guide NIH’s and BARDA’s research and development of MCMs to mitigate chemical, radiological, and nuclear (CRN) threats, and how investments in product or platform development in one area may be leveraged for the other. Although the USG has preparedness plans for many scenarios, the primary focus of this paper will be CRN threats, with occasional discussion of items stockpiled for general medical use or other conditions (e.g., infection, thermal burn) that may also be of use in a CRN response (Table 1).

## Background

### Funding Agencies Overview

Within the HHS, the two main entities that identify and fund promising MCM candidates to address CRN threats are NIAID and BARDA. The thesis of the current effort is that tackling the complexities in biological responses within the CRN threat space can be best addressed with a trans-agency approach. The CRN community of funding agencies has an organized research strategy to (1) establish appropriate nonclinical models representative of the human condition; (2) develop US Food and Drug Administration (FDA)-cleared diagnostics; and (3) advance therapeutics toward FDA approval for inclusion in the SNS to enhance public health emergency preparedness. Collaborative product development and regulatory strategies enable all USG agencies to be smarter and more efficient in accomplishing these goals. Together, NIAID and BARDA support the full spectrum of threat relevant MCM research and development with the goal of advancing products to FDA approval or clearance.

NIAID is the lead institute within NIH for the development of MCMs to mitigate/treat chemical and radiation injuries. The Radiological and Nuclear Countermeasures Program (RNCP), established in 2004 within NIAID, is tasked with developing a robust research program to accelerate the development and deployment of new approaches to assess and treat radiation injuries through grants, cooperative agreements, and contracts, as well as conduct of scientific meetings for its many programs. This mandate involves the development of safe and efficacious MCMs to treat radiation and chemical injuries by funding research to develop products to address injuries to the hematopoietic, gastrointestinal (GI), pulmonary, cutaneous, renal, cardiovascular, ocular (CCRP only), and central nervous systems. In addition, NIAID/RNCP support for research includes the development of decorporation agents targeting internal radionuclide contamination, biodosimetry platforms, and biomarker discovery—capabilities critical to responding to a mass casualty incident involving radiation or nuclear material.

The NIAID CCRP—a collaborative network of academic, industry, and federal laboratories—utilizes the expertise of multiple NIH Institutes and Centers (IC) to manage the research and early development of MCMs against toxic chemical exposure.^1^ This trans-NIH partnership allows the CCRP to capitalize on relevant subject matter expertise in pulmonary, dermal, ocular, and neurological research that already exists at the respective NIH institutes and their extramural communities.^2^ The CCRP at the NIAID is responsible for overall execution of the NIH Strategic Plan and Research Agenda for Medical Countermeasures Against Chemical Threats (www.niaid.nih.gov/sites/default/files/NIH-Strategic-Plan-and-Research-Agenda200708.pdf) and provides over-sight and funding to various R21, R01, R34, UG3/UH3, and Small Business research grant and cooperative agreement initiatives administered by partner ICs, including the Countermeasures Against Chemical Threats (CounterACT) translational program.

BARDA provides an integrated and systematic approach to the advanced development of the necessary vaccines, therapeutics, and diagnostic tools for public health medical emergencies. BARDA was established in 2006 and mandated by Congress to catalyze innovation in advanced research and development, manufacturing, and procurement of MCMs. These lifesaving MCMs are needed to protect people during public health emergencies caused by CBRN incidents (whether accidental or intentional), pandemic influenza, and other emerging infectious diseases. BARDA works closely with interagency partners through the Public Health Emergency Medical Countermeasures Enterprise (PHEMCE) to ensure a coordinated, whole-of-government approach to MCM preparedness and response. BARDA provides end-to-end product development expertise and non-dilutive funding through public-private partnerships with industry, academia, and interagency partners to facilitate advanced development of MCMs.

### Understanding the Challenge

**Figure 1.**
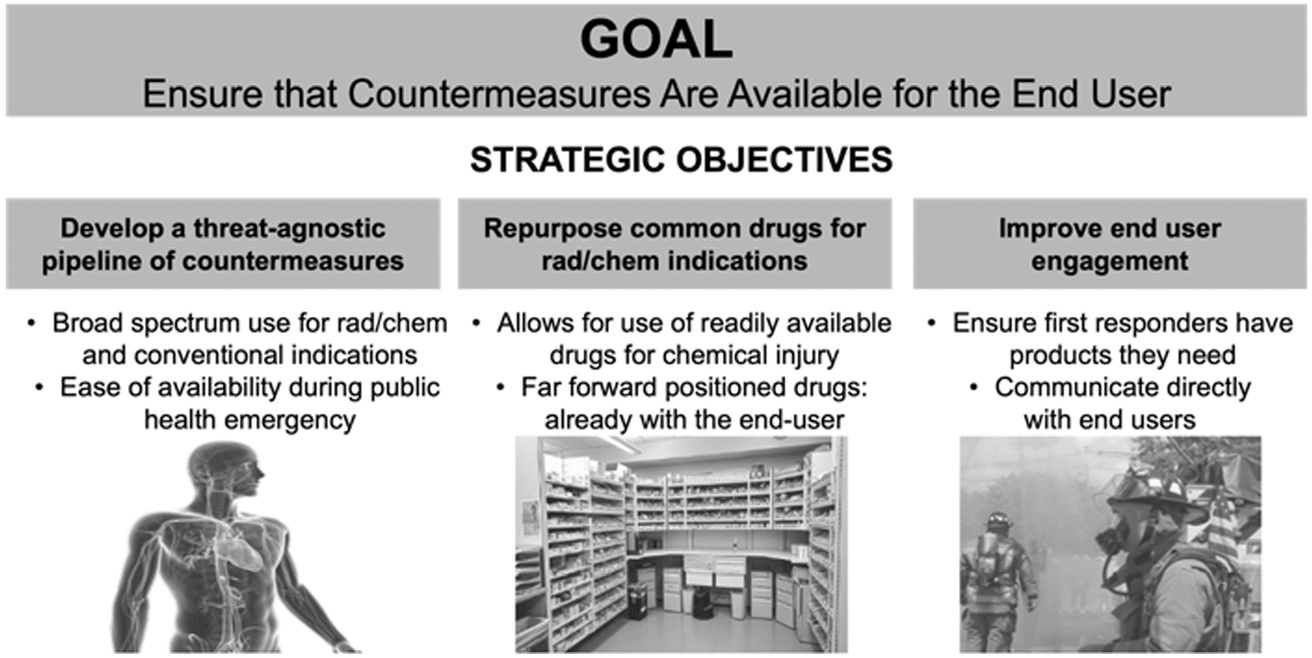
Representative image of the new “threat-agnostic” approach for development and procurement adopted by the chemical MCM branch of BARDA.

With a limited budget to procure medical products that could be needed for a broad range of CRN threats, the USG has moved away from earlier approaches in which products were sought for individual scenarios with narrow indications. In the new development and procurement paradigm, desirable products are threat-agnostic in that they address clinical symptoms/conditions from an exposure, irrespective of the causative agent (Figure 1). For this reason, even though they may be distinctly different in nature, there is an advantage to looking at all CRN threats through a common lens. Exposure to chemical and radiological agents can cause injuries that share symptomology and pathophysiology with commonly seen medical conditions. This “treat the injury, not the agent” approach is cause-agnostic, and it directly aligns with toxidrome identification, a standard practice of emergency medicine, particularly during situations in which there is little time to confirm the initial insult causing the observed symptoms.

### Repurposing Already Approved Products

In collaboration with NIAID’s RNCP and CCRP,^3^ BARDA has a progressive strategy to focus on CRN areas by prioritizing the repurposing of common drugs that already have FDA approval/licensure for another clinical indication where there is a symptomatic overlap from exposure to one or more CRN agents. Repurposing widely stocked, commercially available, and FDA-approved pharmaceuticals (and formulations allowing alternative routes of administration) as potential MCMs has many advantages:

#### Product familiarity for end users

Existing treatments that are used regularly in the clinic ensure that end users are familiar with their administration and possible adverse effects, thus reducing the need for special training.

#### Product access

Known treatments are expected to be readily available in an area where they would need to be used, thus cutting response delays in a time-sensitive situation. Combined with local hospital availability, repurposing reduces the workload for emergency managers to develop complex deployment strategies for stockpiled treatments.

#### Shortened regulatory pathway

Extending the label indication of available therapeutics to include treatment of a CRN injury shortens the pathway to FDA approval because approved products would already have extensive pharmacology and toxicology profiles and robust patient safety and efficacy data.

#### Commercial sustainability

Expanding the availability of the approved product for the CRN indication improves sustainability as well as market opportunity and scalability for industry partners, which are crucial since accessibility of a product is as important as its efficacy.

HHS has had several successes in repurposing products, notably the FDA approval of drugs to increase survival and neutrophil recovery following exposure to myelosuppressive doses of radiation: Neupogen^®^ and Neulasta^®^ (Amgen, supported by NIAID) and Leukine (Sanofi, supported by BARDA). In 2022, another product, Udenyca^®^ (a Neulasta biosimilar), was also approved. In 2023, Stimufend^®^ (pegfilgrastim-fpgk), a second Neulasta biosimilar, was also approved. NIAID and BARDA funded pivotal work that led to approval of Nplate^®^ (Amgen) to address radiation-induced thrombocytopenia. All these products were already approved for treatment of either oncologic or chronic disease conditions. Another successful repurposing effort is the BARDA-supported FDA approval of Seizalam (Midazolam for intramuscular [IM] injection) in 2018 for the treatment of status epilepticus (SE) seizures.

Repurposing is a major tenet of the HHS CRN MCM development strategy. BARDA has several mechanisms that provide opportunities for advanced research and development of FDA-approved and developmental drugs as candidates for CRN MCMs. For instance, the Chem MCM Program supports the Repurposing Drugs in Response to Chemical Threats initiative (ReDIRECT) Area of Interest in BARDA’s EZ-BAA, in collaboration with the BARDA Division of Research, Innovation, and Ventures (DRIVe). ReDIRECT partners with innovators to repurpose commonly available therapeutics to treat conditions resulting from exposure to chemical agents and enable a rapid response. The BARDA Broad Agency Announcement (BAA) funding mechanism also supports the development of repurposed MCMs.

In the chemical therapeutics mission space, the repurposing strategy for the Chemical MCM Program is to support the development of interventions and pharmaceutical candidates to address high priority chemical threats, including opioids (also called *pharmaceutical-based agents*), chlorine, vesicants, nerve agents, and other priority high-risk chemicals listed in the US Department of Homeland Security (DHS) Chemical Terrorism Risk Assessment. Chemical incidents are unpredictable and cause fast-acting injuries for which there are few approved MCMs or diagnostics. Given the short time frame from chemical exposure to injury, coupled with the lack of assays and time for agent identification, clinical treatment of chemical injury is often determined by the acute symptoms following exposure. Therefore, the focus is on developing a strategy that takes advantage of the overlapping symptomology between chemical injuries and common medical conditions. Developing such a product for a CRN indication may be a more cost-effective and time-efficient venture, factors that are especially attractive in the context of MCM development.

### Approaching the Problem

Using a systems biology approach that looks to treat the injury rather than the insult opens the possibility of identifying common research areas within CRN. Cross-referencing available data on the pathologies common to both chemical and radiological insults could reveal unique biomarkers, mechanisms of action, and interventional targets that are currently unknown. Key areas with common threads across CRN include inflammation, apoptosis, vascular injury, sepsis, coagulopathy, fibrinolysis, ischemic injuries, fibrosis, neovascularization, mechanical trauma, thermal burn/skin injury, and secondary infectious disease. Other disease states can also be informative to research efforts. For example, parallel etiologies between coronavirus disease (COVID-19) and irradiation injury implicate specific organ (e.g., vasculature, lungs, heart, kidneys, gut, and brain) and immune system involvement, which should be further explored.^4^ Endothelial damage is observed following both radiological and chemical injury, as are delayed effects persisting months and years in the body’s response to a variety of CRN threats.

Traditionally, USG agencies have focused on studying the biological effects of CRN threats separately, with the goal to treat the biological damage from specific threats. However, in the last 10 years, BARDA and partner agencies have been working together to address CRN injuries. This paradigm shift is, in part, due to an evolving understanding of the pathologies caused by CRN threats. A salient example of this is the impact of a variety of CRN threats on the vascular system. In animal models of acute radiation syndrome (ARS), the hematopoietic system is damaged and vascular injuries occur, which are believed to contribute to multiorgan damage (MOD). Vascular impacts and MOD are seen immediately in the GI and hematopoietic systems and as delayed effects of acute radiation exposure (DEARE) in the lung; these effects in the lung may include pneumonitis and fibrosis. Similarly, exposure to certain chemical agents such as sulfur mustard (SM) induces vascular syndromes and fibrosis in the lung. Sulfur mustard can also induce cytotoxic effects on hematopoietic stem cells at high exposure levels.

### Considering Chemical Threats

One of the main challenges in addressing medical consequences of exposure to chemical agents is the very short time from exposure to injury, which can occur in minutes to hours. Although several platforms are available to discern the identity of various chemicals, for example, the CDC Rapid Toxic Screen (RTS), which can identify up to 150 chemical agents in blood and urine samples,^a^ these technologies are not expected to be useful due to the extended time required for sample preparation and analysis during and/or immediately after high consequence, public health emergencies involving mass casualties. Consequently, the clinical management of chemical injury is often dictated by the acute toxic symptoms presented following exposure, that is, toxidromes. The BARDA Chemical MCM Program and the NIAID CCRP have developed a strategy that takes advantage of the overlapping symptomology between chemical injuries and more common medical conditions (e.g., acute lung injury, acute respiratory distress syndrome, pulmonary fibrosis, and uncontrolled seizures/convulsions). The DHS has identified nearly 200 highly toxic chemical compounds (HTCs) as credible public health security threats. Many of these chemical threats are easily accessible and widely available due to the large number of manufacturing sites, general commercial use, and extensive transportation across the nation. Given the wide availability of chemical agents combined with the general lack of effective therapeutics, it is imperative that new MCMs be developed to address the adverse acute and long-term health effects caused by exposure to chemical agents.

To best address the broad chemical threat spectrum, the USG has adopted an approach that aligns with toxidrome identification, as is standard practice in emergency medicine. The identified HTCs cause injuries primarily within five priority toxidromes: neurologic (e.g., organophosphate chemical warfare nerve agent sarin, soman, and VX), pulmonary (e.g., sulfur mustard, chlorine, phosgene), respiratory (e.g., synthetic opioids fentanyl and carfentanil), metabolic (e.g., cyanide), and vesicating (e.g., sulfur mustard and Lewisite). Diagnosis of these toxidromes allows for assessment and treatment of chemical injuries without the need to identify the chemical agent causing the injury. To this end, the NIAID CCRP and BARDA’s Chemical MCM Program are focused on discovery and advanced development of therapeutics geared toward treating symptoms associated with chemical exposure, rather than “antidotes” for specific chemical agents. This toxidrome-based approach allows for broad-spectrum therapeutic utility, while aligning with conventional practice of medical toxicology. The strategy of using products to treat chemical injury that are already FDA approved, or are being developed for common clinical indications, provides opportunities to develop multi-purpose, broad-spectrum candidates that would be readily available to emergency responders where and when they need them. The “treat the injury not the agent” paradigm has driven the Chemical MCM Program’s investments in capabilities for repurposing commonly used products for chemical indications. The existing national stockpile is focused on capabilities to treat specific chemical incidents (e.g., CHEMPACK for nerve agent release); however, their supply is limited, and distance may delay the timely delivery of lifesaving MCMs to the scene of a chemical incident. With chemical MCMs especially, prepositioning therapeutics throughout the country can reduce the time to delivery of potentially lifesaving medical treatments, but this approach also imposes logistical and planning burdens. Instead, being able to use an already FDA-approved and readily available product could potentially increase access to a MCM during an emergency, as more prehospital first responders with varying levels of training and certification may be able to administer such treatments and thus streamline the response.

### Radiation Skin Injuries

In addition to the shift in thinking discussed above, BARDA continues to engage with the FDA/CDRH to revisit the nomenclature associated with radiation injuries to skin and redirect product development strategies. Whereas mild injuries to the skin, a common side effect seen in patients undergoing cancer radiation therapy, are termed *radiation dermatitis (RD),* the term for skin injuries from accidental exposure to the skin, arguably from larger exposures, is *cutaneous radiation injury (CRI).* The origin of these terms either from clinical use or animal model research has played a role, and now a broader recognition of the need to reconcile terminologies has been proposed by BARDA and others.^5^ This approach extends the application of the MCM to clinical conditions seen in routine health care like RD. A direct benefit would be bolstering the market sustainability and product access for use in mitigation of injuries from nuclear fallout exposure to the broader population without the need for stockpile and distribution. In 2022, BARDA’s partner Argentum was successful in seeking the first in class FDA clearance for their Silverlon product for limited CRI conditions and RD. This paradigm shift in building preparedness by BARDA will achieve its full potential with expanding the indication for use of Silverlon over the full range of CRI. BARDA’s first investment was to procure Silverlon for management of burn injuries in the field. BARDA supported an expanded indication for management of skin injuries from SM in 2019. Continued investment and development through 2022 led to its indication for limited use in CRI and RD,^b^ as discussed above. The 2022 US FDA approval of MediWound’s NexoBrid was another BARDA-led effort. NexoBrid is an enzymatic debridement agent that was already approved in several countries, indicated for eschar removal in adults with deep partial thickness and/or full thickness thermal burns.^c^ Similarly, KeraNetics’ KeraStat^®^ topical cream received FDA clearance in 2020 for management of thermal burns and radiation dermatitis,^d^ and BARDA is supporting additional efforts to expand the indication for use in CRI. This multi-purpose, broad-spectrum approach has proven to be cost-effective in preparing for mass casualty events involving CRN agents.

### Radiation Vascular Injuries

RNCP’s and BARDA’s recognition that vascular injury is a potential cause of multiorgan injury in ARS and DEARE continues to drive development of MCMs by both programs^6^ to mitigate vascular damage. Due to the similarity in injury among radiation, SM, and certain other chemical agents, some of these vascular MCMs are also being tested for use in chemical injury models. To enhance understanding of vascular biology and, in particular, coagulation in the context of trauma, NHLBI and DoD established TACTIC (Trans-Agency Consortium for Trauma-Induced Coagulopathy). This effort focuses on the similarities between traumatic mechanical injury and radiation injury. The 12 basic science projects conducted by 21 investigators led to the understanding that vascular injury due to radiation mirrors vascular dysfunction observed in trauma patients. Endotheliopathy, coagulopathy, and inflammation are observed in traumatic injury as well as in radiation injury and contribute to the observed multiorgan injury.^6^ As in radiation injury, endothelial damage is a wide-reaching pathology that is observed in response to a variety of CRN threats, including SM.^7^

### Addressing Microbial Threats

Another approach centers around the prophylactic use of antibiotics to protect victims of a nuclear event. While significant infections can be mitigated by antibiotics, a segment of patients would likely experience antimicrobial-resistant infections. To mitigate this risk, it is useful to study not only what antibiotics could prevent mortality, but also determine whether those antibiotics would have limited use due to resistant bacteria. Such modeling could be useful for any threats that could cause widespread bacterial infections or require antimicrobial treatment. BARDA has established a joint program between its Antimicrobial and Radiological/Nuclear branches to further examine the role that various antibiotic classes may play in the treatment of infections as a result of radiation injury.

### Emergency Response Logistics

A primary focus of ASPR is development of best practices for emergency response. To support this mission, BARDA sponsored research to develop and optimize an evidence-based protocol for mass-casualty decontamination following a chemical incident. The Primary Response Incident Scene Management (PRISM) guidance addresses strategic, tactical, and operational aspects of preparedness and response to mass-casualty incidents involving the deliberate or accidental release of hazardous materials, including chemical warfare agents.^e^ Some of the same decontamination principles could likely be applied following exposure to radiological agents. PRISM provides a simple-to-follow protocol for disrobing and decontamination that is faster and more effective than current processes, which vary from locality to locality across the United States. One notable finding of the research is that carefully removing clothes and wiping skin with a paper towel or absorbent cloth can remove more than 99% of chemical contamination. In addition, data from *in vitro* and human volunteer studies suggest that hair offers a protective effect from chemical exposure in the short-term, yet it may retain chemicals longer than the skin. It is possible that this could also apply to radioactive particle contamination. The findings also point to the need to avoid the common practice of using high-pressure water from fire engines to shower patients who are still clothed, as showering in contaminated clothing washes chemicals through clothing and into the skin. Based on the PRISM recommendations, community planners can build scientifically sound actions into emergency response plans.

In addition, to aid first responders with critical decisions regarding decontamination following a chemical incident, BARDA supported the development of a mathematical decision-aid tool for chemical decontamination named *Algorithm Suggesting Proportionate Response Engagement (ASPIRE).* ASPIRE, which is based on the PRISM Guidance, helps first responders assess the utility of disrobing and decontamination on a case-by-case basis. ASPIRE can be found in Chemical Hazards Medical Management (CHEMM) website^f^ and the Wireless Information System for Emergency Responders (WISER) phone app.^g^ Much of this knowledge gained in consideration of chemical exposures is likely to be useful following a radiological or nuclear incident, but further research is needed to establish the exact parameters.

Another critical resource to prepare and respond to public health emergency is ready access to accurate information. BARDA supports the Radiation Emergency Medical Management (REMM) tool (previously maintained by the National Library of Medicine [NLM]).^h^ REMM is a website developed by HHS/ASPR that provides guidance for a range of health care providers regarding the clinical diagnosis and treatment of radiation injuries, as well as just-in-time information to those without formal radiation medicine expertise. REMM houses the Exposure and Symptom Triage (EAST) tool to assess radiation exposure after a nuclear detonation, triage guidelines, and algorithms for radiation exposure and contamination treatment under a variety of resource settings. REMM is a preparedness and response tool for the broad medical community. ASPR and BARDA also sponsored the development of the CHEMM tool, in cooperation with the NIH/NLM and subject matter experts in medicine, emergency response, and toxicology. The goal of the CHEMM tool is to enable first responders, first receivers, health care providers, and planners to plan, respond, recover, and mitigate the effects of incidents involving chemicals. The platform is comprehensive, user-friendly, web-based, and downloadable, allowing for its use during an event, even when the Internet may be unavailable.

## Current CRN Environment

### Status of Radiation/Chemical Preparedness—Concepts of Operation (CONOPs) and the SNS

Six drugs are currently approved by the FDA for the treatment of myelosuppressive doses of radiation. Five products are leukocyte growth factors that target the myeloid compartment to increase neutrophil and/or macrophage counts post-irradiation. In 2021, the FDA approved a megakaryocyte-targeted product to increase platelet counts post-irradiation. Given their approval status, these products are available, can be transported across state lines, and can be used by health care personnel during a radiological public health emergency without further need for approvals such as an Emergency Use Authorization (EUA). They could also be utilized as a vendor- or end user-managed inventory, where the US Government provides funding for a “stock bubble” to be held by the manufacturer,^8^ or at hospitals, pharmacies, or on ambulances, etc.^9^ Decorporation and blocking agents to address internalized radionuclides are also approved and available. These include Prussian blue, calcium and zinc diethylenetriaminepentaacetic acid (DTPA), and potassium iodide. However, these products are not necessarily expected to be widely distributed or in regular use in the health care community and will require deployment in the event of a radiological incident.^i^ NIAID and BARDA supported efforts to improve the utility of these agents, including (1) development of oral formulations of DTPA^10-12^; (2) studies to increase potency and range of radionuclide binding of novel agents^13^; and (3) evaluation of formulations of Prussian blue that are more readily used by special populations (eg, children and older adults).^j^

Given that the timelines for injuries in most chemical emergencies are much shorter than those anticipated for a radiological or nuclear crisis, the CHEMPACK program began as an SNS initiative in 2003. The CHEMPACK program is envisioned as a comprehensive capability for the effective use of MCMs in the event of an attack on civilians with nerve agents. The program builds upon the existing emergency response system by adding education, training, and exercise components, and by pre-positioning of antidotes throughout strategic locations around the country. These strategic locations are selected by local authorities to support a rapid hazardous materials (HAZMAT) response by allowing quick access to lifesaving MCMs by first responders and hospital personnel in the event of a nerve agent incident.

### Crossing Mission Spaces—MCMs

Some radiological and nuclear MCMs may be of benefit to the chemical threat space as well. Some chemicals, specifically SM, can cause injury to the bone marrow.^14^ For this reason, studies were done to determine whether leukocyte growth factors could also have a positive impact in addressing SM-induced myelosuppression. In nonclinical studies, G-CSF injection 30 minutes after SM exposure led to improved survival and mitigated loss of white blood cells.^15^ In addition, increased levels of G-CSF^16^ or GM-CSF^17^ were identified in bronchoalveolar lavage fluid taken from patients with lung fibrosis following mustard gas exposure even decades later, suggesting a possible role for neutrophils in the etiology of the disease and, by extension, potential use of leukocyte growth factors as a therapeutic in lung fibrosis. Also, SM exposure can lead to increases in macrophage and neutrophil levels in exposed skin and concomitant elevated expression of G-CSF levels.^18^

Although not yet approved by the FDA for a radiation indication, there are other widely available compounds (either products approved for other diseases states or nutraceuticals) that could be beneficial for both radiation and chemical indications. These include therapeutics targeted toward skin injuries, for example, curcumin,^19-22^ N-acetylcysteine (NAC),^23-26^ and various COX-2 inhibitors.^27-30^ NAC has also been found to mitigate/treat SM-^31^ and radiation-^32^ induced lung injury in humans. Other compounds like KL4, a surfactant approved for respiratory distress in neonates, have shown promise for radiation-induced lung damage,^33^ with potential for use in SM exposures as well, based on the reported efficacy of other surfactants.^34,35^ Similarly, products indicated for idiopathic pulmonary fibrosis (IPF), including drugs that target pathways known to be involved in fibrosis (e.g., transforming growth factor β, PDGFR α and β; FGFR, VEGFR, tyrosine kinases), could be used for both radiation and SM indications to interfere with the lung injury cascade. Both nintedanib (Ofev) and pirfenidone (Esbriet) are approved for use in IPF and are in testing for the radiation indication (National Clinical Trail #00020631).^36,37^ These or similar products may have the potential to mitigate SM-induced lung fibrosis.^38^

Drugs like angiotensin-converting enzyme (ACE) inhibitors, which block elements that contribute to hypertension as their primary mechanism of action, were shown to reduce lethality associated with radiation exposure (both hematopoietic,^39^ and from late lung complications).^40,41^ It is conceivable that this product could also find use in the mitigation of certain chemical-induced lung complications. Still other products in advanced development for radiation-induced dermal^42^ or lung^43^ indications could be considered for potential use in an SM exposure scenario, and those under study for SM could be applied to the radiation space.^44,45^ Additional products of interest to both missions are detailed elsewhere in this special issue. It is critical that researchers working in development of MCMs for either chemical or radiation exposures be aware of work done in the other areas in order to apply findings across both threat spectrums.

### Response Timelines

Requirements for use of products to address radiation-induced injuries allow for some response time, with many experts suggesting that distribution and/or administration will likely take at least 24 hours.^46^ For this reason, stockpiling represents an acceptable means of making these products available for some threats^47,48^; however, further pre-positioning in both vendor- and end user-managed paradigms is a preferred strategy for faster-acting threats.^46^ It may also be possible to use items that are already approved for other traditional clinical indications and may be available during a public health emergency, such as items carried by first responders, or those maintained in hospitals and/or local pharmacies. There are, for example, certain standard-of-care approaches like antibiotics, resuscitative fluids, and broad-spectrum skin care products with overlapping uses.

Pre-positioning for a radiation incident may be limited when it comes to FDA-approved MCMs. While these MCMs are in use at hospitals and other medical settings, they are not regularly carried or administered by first responders. However, that is not the case for blood products. Blood products are forward deployed in a variety of settings, including by first responders in civilian settings and by soldiers for battlefield use. BARDA continues to support research into the use of blood products to increase survival in patients with ARS and trauma and to invest in the development of next-generation blood products with increased storage lifespan and shelf-stability. These products are intended for use not only by trauma doctors, but also by first responders. Such blood products would benefit not only ARS patients, but also patients with mechanical injuries expected during a nuclear incident.

### Breadth of the Burn-Radiation-Chemical MCM Research Communities—Overlaps

BARDA encourages its product developers to explore both the radiation and chemical domains when applicable. A demonstratable result of this strategy is reflected in advanced development of the burn contact wound dressing, Silverlon, mentioned above, as an MCM that is FDA cleared or approved for treatment of SM burns, CRI, and RD (described earlier). Silverlon is a commercially available silver-plated nylon dressing indicated for the management of first- and second-degree thermal burns. In 2019, Silverlon became the first ever FDA-approved treatment for SM wounds. BARDA’s support for the SM indication began in 2013, and, in 2022, the product was cleared as a device for treating CRI resulting from a public health emergency.^k^

## Research Strategies and Methods

### Research Models for MCM Discovery

Research involving radiation and chemical threats is reliant on the use of animal models to simulate the biological effects of these insults in humans. Understandably, due to the ethical nature of conducting such studies in humans, research in this area often follows the FDA Animal Rule licensure pathway (described in detail below).^49^ The Animal Rule generally requires proof of MCM efficacy in at least one animal model predictive of the human response; however, this is widely considered to mean one small animal and one large animal model. For this reason, BARDA actively supports non-clinical model development in several species. Rodents are often the primary species utilized in early preclinical studies before moving onto higher-order species for more advanced work. Typically, total body irradiation (TBI) is used to model the expected hematopoietic injuries after a radiological or nuclear incident, and the radiation doses commonly used result in a mortality of 50% (LD_50_) over a period of 30–60 days, depending on the model. Other models of radiation injury include partial body irradiation (PBI). PBI models typically involve larger doses of radiation to a specific organ (such as whole thorax lung irradiation [WTLI]) or involves sparing a portion of bone marrow (BM) to prevent total myelosuppression. PBI experiments are performed to model organ-specific injuries, with BM sparing models being used to permit the study of multiple organ injury induced at higher doses of radiation than would be survivable using TBI models. Using these PBI models, researchers have identified a variety of DEARE injuries, such as radiation-induced lung injuries (RILI), which encompass pneumonitis and fibrosis.

The TBI mouse animal model allows for the study of hematopoietic (H)-ARS. H-ARS can include prolonged immunosuppression, impaired function of hematopoietic stem cells, and multiorgan DEARE in long-term survivors of TBI doses.^50-58^ Included within the murine H-ARS modeling repertoire are young adult,^57,59-62^ outbred,^56^ pediatric,^58^ and geriatric^63^ mice that have been instrumental in efficacy studies for the development of MCMs. However, murine models have limitations. Mouse models have limited capability for longitudinal blood collection, given the blood volume of a mouse. The radiosensitivity of mice is highly strain-dependent, and the mouse kinetics of H-ARS differs from humans. Therefore, BARDA and NIAID supported the development of larger animal models, including minipig and New Zealand white rabbit. These large animal models for longitudinal blood sampling mimic human ARS in terms of onset, symptoms, and blood kinetics.

The high sensitivity of the lung to irradiation and the unique nature of delayed pathology have made this tissue a focal point of DEARE research, with implementation of animal models that have greatly evolved over time. A surrogate model of RILI that is frequently used is the DNA-damaging chemotherapeutic drug bleomycin. However, chemical radio-mimetics can trigger more immediate cell damage in the lung, thus leading to an accelerated form of radiation injury that may not exhibit the more latent development of lung damage observed after radiation exposure.^64^ Researchers pursued local exposure models that exhibit lung damage most reflective of what can be expected in humans. These models with localized lung exposure include the WTLI model and the “top-up” model that involves TBI followed by an addition exposure of WTLI. Most recently, PBI models have been investigated where 2.5 to 8% of the bone marrow is shielded.^43^ These analyses led researchers to develop more refined animal models with the potential to carefully evaluate the efficacy of MCM candidates.

For development of animal models to evaluate chemical threat injury, the BARDA Chemical Countermeasures Program has partnered with the BARDA’s Division of Nonclinical Development (DNCD) to establish animal models of chlorine gas exposure. These models (mouse and swine) serve multiple purposes, including screening novel products, supporting ReDIRECT, providing proof-of-concept data to industry partners, and performing follow-up analyses on promising candidates. While the data generated from these models may be proprietary, the methods used to generate these models will be published in peer-reviewed journals. Models for SM toxicity have also been developed and utilized as publicly available screening resources through partnerships with DNCD and the NIAID CCRP.^65,66^ To accelerate the development of chemical MCMs, the Chemical MCM Program is investing in innovative and enabling technologies for the identification and investigation of promising potential MCMs. Some of these technologies include organ-on-a-chip platforms and artificial intelligence-driven screening to ensure the rapid deployment and prompt administration of life-saving treatments. Applying cutting-edge technology in organ-on-a-chip platforms and computational software for machine learning will support current screening capabilities and give us new strategies for identifying and developing new and effective MCMs for chemical threats. Another area of interest is the development and manufacturing of novel drug delivery technologies for rapid administration of MCMs. The goal is to expand the industrial base for existing technologies, like autoinjectors, while promoting competition for future development of new technologies and reducing reliance on sole-source suppliers.

NASA established an interagency initiative to develop extended longevity tissue chips, partnering with NIAID/RNCP, BARDA, FDA, NIH/National Center for Advancing Translational Sciences (NCATS), and NIH/National Cancer Institute (NCI). This 2022 initiative enables investigation into the natural history of various agency-relevant stressors, such as radiation and the resulting sequelae of ARS. BARDA has additional partnerships with NCI and FDA for the development of organ-on-a-chip models of ARS, as well as funding opportunities through the BAA. The use of these technologies will deepen our understanding of the impact of radiation damage on endothelial and vascular function and on specific tissues or organ systems and enable new MCM development. To replicate human CRI, porcine models are preferred due to the similarities between human and porcine skin. Our close interactions with CDRH led us to hone the development of robust CRI porcine models for the evaluation of two different MCMs under partnership with BARDA.^67^

### Biomarkers, Diagnostics, and Other Approaches

Assays and devices to assess exposure to either radiation or chemical threat agents are of high research and development interest. The CDC developed the RTS, which has the capacity to identify over 150 chemical agents.^l^ Although as of this writing, there is no FDA approved diagnostic device for radiation,^68^ the dicentric chromosome assay is considered the “gold standard” to assess radiation exposure dose.^69^ Rather than the traditional, pre-defined threat approach, researchers propose identifying injury biomarkers by focusing on threat-agnostic biodefense.^m^ Hence, irrespective of the insult (chemical, radiological, or biological), researchers focus on clinically measurable biomarkers that can predict the outcome of the exposure, without prior knowledge of the agent. Biomarker signatures will likely include common patterns of pathologies and will be identified using integrated multi-omics data and imaging modalities to reflect dysfunction in the affected host.

While there are no approved biodosimeters for radiation or chemical threats, there are assays that could be deployed during such an emergency. Available methods for detecting the biological impacts of chemical and radiation exposure are complete blood counts with differentials, which indicate cytopenias that can correlate to exposure level. Other existing methodologies under study for possible future use include computed tomography (CT) and magnetic resonance imaging (MRI) scanning (e.g., of the lung), functional imaging, clinical symptomology, multi-omics signals, and assessment of host-responses to an insult. While these tools and concepts, which could be exploited to develop a predictive signature, are promising concepts, the technology is immature, and regulatory agencies will require a clear correlation between the insult and the pathology.

## Critical Pathways to Approval/Licensure/Clearance—Regulatory Strategies

### FDA Requirements

Establishment of the FDA Animal Rule (AR) in 2002 allowed for approval of new drugs when human efficacy studies were not ethical or feasible for drugs (SubpartI-21, CFR Parts 314.6000.650) or biologics (Subpart H-21, CFR Parts 601.90–95). These regulations specifically addressed the regulatory gaps in MCM development to mitigate serious adverse health events, resulting from exposure to lethal or permanently disabling CBRN threats. The AR approval relies on adequate and well-controlled studies in relevant animal models to determine efficacy of the MCM as well as extensive safety studies in human volunteers. The foundational pillars of the AR necessitate (1) a well-understood pathophysiology sequelae of the insult/injury (chemical, biological, radiological) and its resolution by the MCM, (2) efficacy in more than one animal model predictive of response in humans, (3) study endpoints related to desired benefits in humans (reduction in major morbidity or mortality), and (4) pharmacokinetic (PK)/pharmacodynamic (PD) assessments in animal models and humans that allow for appropriate MCM dose scaling.

### Milestones for Improving US Preparedness

Pyridostigmine bromide was the first MCM approved under the AR in 2003 as a prophylactic against Soman nerve agent poisoning. Since then, FDA’s Center for Biologics Evaluation and Research (CBER) and Center for Drug Evaluation and Research (CDER) have approved over a dozen MCMs under the AR.^n^ However, given unforeseen circumstances, when there is an urgent need for medical interventions and little to no time to conduct elaborate studies to meet the AR requirements, how can first responders and CONOPs planners utilize the existing approved MCMs to better prepare for an emergency response? What are the regulatory means to facilitate a rapid response to such an emergency? How can funding agencies connect policy and regulatory requirements with established scientific norms? These are topics for continued and frequent dialogues.

In the event of a public health emergency, assuming a broad-brush approach to focus on treatment of the symptoms and injury might be the logical next step. For instance, with five MCMs approved to treat radiation-induced immune cell loss, and given the impact of SM exposure on the bone marrow,^70^ it may be possible to use these products for the treatment of SM-induced hematological damage. Preliminary studies on the efficacy of filgrastim in mitigating SM-induced hematological deficits indicate that such a treatment is promising (see Beske *et al.* elsewhere in this special issue). However, to date, there are no FDA-approved MCMs for the treatment of SM-induced hematological toxicity.^66^ Hence, it may be logical to extrapolate the data for radiation-induced hematological deficits to expedite approval of the same MCM for SM-induced hematological deficits. It also may be possible to streamline regulatory processes given the prior approval for the treatment of radiation-induced neutropenia or thrombocytopenia.

This cross-usage may not be applicable in all cases. Preclinical data are crucial to provide evidence of utility. For instance, keratocyte growth factor (KGF) protects mice from radiation-induced GI injury when administered prior to exposure^71^; however, when KGF was administered 24 hours post-irradiation in mice, it did not afford any survival benefit [Satyamitra, unpublished data], and in irradiated NHPs, its use post-irradiation led to worse outcomes.^72^ Similarly, stem cell factor (SCF) improved mouse survival following exposure when administered 8 hours prior to total body irradiation^73^; however, when administered 24 hours post-irradiation, earlier mortality of irradiated mice was observed [M. Satyamitra, unpublished data]. When a completely different toxic insult is used, it is impossible to predict if a threat-agnostic approach would do more harm than good. Hence, some preclinical studies are needed to bridge treatment strategies for different insults.

The benefits and challenges of repositioning or repurposing products to address medical consequences following a chemical or radiological public health emergency have been previously highlighted.^3,74^ Neupogen, Neulasta, Nplate, Udenyca, and Stimufend are approved drugs that were repurposed for acute radiation syndrome. All five MCMs were reviewed by CDER, FDA, and it took 15+ years of planning, research and development, and voting by an advisory council for FDA to approve the first of them (Neupogen) for radiation use. However, based on precedent, Nplate required just 5 years to obtain FDA approval, and no advisory council was convened. Similarly, as previously mentioned, Silverlon, a wound dressing widely used for traumatic injuries, is now FDA-cleared for treatment SM-induced vesicant injuries (2017)^o^ and, more recently, for radiation dermatitis and CRI.^p,q^

A widely available drug indicated for the treatment of common medical conditions for injuries caused by a CRN agent is Seizalam (Midazolam for intramuscular [IM] injection), which received FDA approval in 2018 for the treatment of status epilepticus (SE) seizures, including those caused by exposure to nerve agents. This approach departed from the more conventional regulatory approval for a New Drug Application (NDA)/Biologics License Application (BLA) for a specific CRN indication (e.g., nerve agent-induced seizures), rather focusing on pursuing regulatory MCM approval based on the general symptoms observed to treat a specific symptom (e.g., SE seizures), independent of the cause of the initial injury (e.g., nerve agents, epilepsy). The approval was based on abundant evidence showing that SE caused by nerve agents and organophosphate pesticides poisoning is fundamentally identical to SE precipitated by other causes. Based on this work, BARDA supported the advanced research, development, and procurement of midazolam vials. The FDA agreed that IM midazolam for acute treatment of generalized convulsive SE (GCSE) should not be limited to nerve agent-induced SE, rather approved for the treatment of all presentations of GCSE, leading to Seizalam’s 2018 approval for the treatment of SE. This approach aligns with the “treat the symptom, not the agent” strategy, which positions widely used treatments for common medical conditions to be available for use by first responders or medical professionals for use following exposure to a chemical threat. Applying this successful approach to the development of MCMs for CRN threat areas could result in a streamlined regulatory pathway and wide availability of therapeutics routinely used for the treatment of common medical conditions to address injury caused by exposure to CRN agents.

Another widely available therapeutic being repurposed for the treatment of chemical exposure is atropine. Atropine is an anti-muscarinic drug used, among other indications, to treat organophosphorus nerve agent poisonings, symptomatic bradycardia, and to dilate pupils for posterior chamber eye examination. Atropine can be administered IM, intravenous [IV], or into the eye. Atropine ophthalmic drops have been administered sublingually (SL) in several off-label clinical settings for conditions such as excessive salivation and for easing the final moments in hospice patients. BARDA supported a Phase 1 clinical trial to evaluate the bioavailability and pharmacokinetics of sublingually administered atropine sulfate ophthalmic solution compared to the IV route of administration (NCT04290039). Findings suggest that sublingual atropine is safe and well tolerated. Bioavailability of sublingual atropine was determined to be ~63% of that achieved via IV administration. Bioequivalence of SL vs IM would be expected to reach 80–120% bioavailability, but studies remain to be conducted. In patients suffering mild organophosphate intoxication symptoms, SL administration of the ophthalmic formulation of atropine may be readily available to treat their symptoms, while currently stockpiled atropine autoinjectors and multidose vials could be preserved for the subset of patients experiencing severe or life-threatening poisoning.

## Conclusion

In preparing the nation to respond to CRN threats, a more efficient approach than purchasing and stockpiling of products is imperative. Implementation of a “threat agnostic” approach that focuses on biological outcomes following CRN injury may increase preparedness. Key to this strategy is the repurposing of FDA-approved products, including medical devices and resources such as those involved in standard medical care (e.g., fluids, dressings), for a CRN indication. Priority should be given to widely available and widely used FDA-approved products to ensure product viability and sustainability. The USG strongly suggests that developers pursue generic commercial indications for their products, in addition to indication(s) for CRN threats. Having proven treatments and resources available that are familiar to the medical community, along with accessible, accurate, and timely information, paves the way for a robust US preparedness posture and an efficient multi-use and threat-agnostic medical response.

## Figures and Tables

**Table 1.** Areas of overlap between the radiation and chemical threat domains within the HHS

RADIATION & CHEMICAL THREATS – OVERLAPPING DOMAINS		
Concepts of Operations Post-Exposure, Stockpile Timelines, Multi-Utility Scarce Resources	Scientific Approaches Biomarkers (Triage, Patient Management, Prognosis, Efficacy) Mechanisms of Action (Targets, Treatments)	Regulatory FDA Animal Rule Repurposing Human Safety
Disease States Acute/Chronic Fibrosis, Inflammation Oxidative Stress	Animal Models Rodents Large Animals (NHP, Swine, Canine)	Organs Lung, Skin Bone Marrow Central Nervous System

## Footnotes

a
https://www.cdc.gov/biomonitoring/chemical_threat_agents.html

b
https://www.accessdata.fda.gov/scripts/cdrh/cfdocs/cfpmn/pmn.cfm?ID=K221218

c
https://www.accessdata.fda.gov/drugsatfda_docs/label/2023/761192s000lbl.pdf

d
https://www.accessdata.fda.gov/cdrh_docs/pdf19/K192386.pdf

e
https://www.medicalcountermeasures.gov/barda/cbrn/prism/

f
https://chemm.hhs.gov/

g
https://wiser.nlm.nih.gov/

h
https://remm.hhs.gov/

i
https://www.cdc.gov/nceh/radiation/emergencies/countermeasures.htm

j
https://www.phe.gov/Preparedness/news/Pages/prussianblue.aspx

k
https://www.accessdata.fda.gov/scripts/cdrh/cfdocs/cfpmn/pmn.cfm?ID=K221218

l
https://www.cdc.gov/biomonitoring/chemical_threat_agents.html

m
https://www.pnnl.gov/threat-agnostic-biodefense

n
https://www.fda.gov/media/150191/download

o
https://www.accessdata.fda.gov/scripts/cdrh/cfdocs/cfpmn/pmn.cfm?ID=K190343

p
https://www.accessdata.fda.gov/scripts/cdrh/cfdocs/cfpmn/pmn.cfm?ID=K221218

q ~https://www.prweb.com/releases/silverlon_receives_fda_breakthrough_device_designation_for_radiation_dermatitis_and_cutaneous_radiation_injury/prweb18362909.htm#:~:text=Argentum%20Medical%2C%20a%20medical%20device%20leader%20and%20pioneer,its%20Silverlon%20%C2%AE%20Wound%20Contact%2C%20Burn%20Contact%20Dressings.

## References

[1] Yeung DT, Harper JR, Platoff GE. The National Institutes of Health Chemical Countermeasures Research Program (NIH CCRP): a collaborative opportunity to develop effective and accessible chemical medical countermeasures for the American people. Drug Dev Res. Published online June 22, 2020. doi: 10.1002/ddr.21707 PMC7752830 32573018

[2] Yeung DT, Harper JR, Platoff GE. Supporting fundamental chemical toxicology research to inform medical countermeasure developments: The National Institutes of Health Chemical Countermeasures Research Program. Chem Res Toxicol. 2020;33(4):855–859. doi: 10.1021/acs.chemrestox.0c00086 32196324 PMC7174080

[3] DiCarlo AL, Cassatt DR, Dowling WE, Challenges and benefits of repurposing products for use during a radiation public health emergency: lessons learned from biological threats and other disease treatments. Radiat Res. 2018;190(6):659–676. doi: 10.1667/rr15137.1 30160600 PMC8466052

[4] Rios CI, Cassatt DR, Hollingsworth BA, Commonalities between COVID-19 and radiation injury. Radiat Res. Published online October 2020. doi: 10.1667/RADE-20-00188.1 PMC7861125 33064832

[5] Burnett LR, Hughes RT, Rejeski AF, Review of the terminology describing ionizing radiation-induced skin injury: a case for standardization. Technol Cancer Res Treat. 2021;20:15330338211039681. doi: 10.1177/15330338211039681 34613833 PMC8504211

[6] Satyamitra MM, DiCarlo AL, Taliaferro L. Understanding the pathophysiology and challenges of development of medical countermeasures for radiation-induced vascular/endothelial cell injuries: report of a NIAID workshop, August 20, 2015. Radiat Res. 2016;186(2):99–111. doi: 10.1667/rr14436.1 27387859 PMC5050526

[7] McGraw MD, Osborne CM, Mastej EJ, Editor’s highlight: pulmonary vascular thrombosis in rats exposed to inhaled sulfur mustard. Toxicol Sci. 2017;159(2):461–469. doi: 10.1093/toxsci/kfx151 28962529 PMC5837673

[8] Hanfling D. Equipment, supplies, and pharmaceuticals: how much might it cost to achieve basic surge capacity? Acad Emerg Med. 2006;13(11):1232–1237. doi: 10.1197/j.aem.2006.03.567 16801633

[9] Coleman CN, Hrdina C, Casagrande R, User-managed inventory: an approach to forward-deployment of urgently needed medical counter-measures for mass-casualty and terrorism incidents. Disaster Med Public Health Prep. 2012;6(4):408–414. doi: 10.1001/dmp.2012.46a 23241473

[10] Shankar GN, Potharaju S, Green CE. Evaluating the toxicity of novel Zn-DTPA tablet formulation in dogs and rats. Drug Dev Res. 2014;75(1):37–46. doi: 10.1002/ddr.21165 24648048

[11] Huckle JE, Sadgrove MP, Leed MG, Synthesis and physicochemical characterization of a diethyl ester prodrug of DTPA and its investigation as an oral decorporation agent in rats. AAPS J. 2016;18(4):972–980. doi: 10.1208/s12248-016-9916-z 27106838

[12] Cassatt DR, Kaminski JM, Hatchett RJ, Medical countermeasures against nuclear threats: Radionuclide decorporation agents. Radiat Res. 2008;170(4):540–548. 19024661 10.1667/rr1485.1 PMC8379839

[13] Abergel RJ, Durbin PW, Kullgren B, Biomimetic actinide chelators: an update on the preclinical development of the orally active hydroxypyridonate decorporation agents 3,4,3-LI(1,2-HOPO) and 5-LIO(Me-3,2-HOPO). Health Phys. 2010;99(3):401–407. doi: 10.1097/HP.0b013e3181c21273 20699704 PMC2921233

[14] Anderson DR, Holmes WW, Lee RB, Sulfur mustard-induced neutropenia: treatment with granulocyte colony-stimulating factor. Mil Med. 2006;171(5):448–453. doi: 10.7205/milmed.171.5.448 16761898

[15] Cai Y, Ma Q, Zhang L, [Therapeutic effects of rhEPO, rhG-CSF on sulfur mustard induced toxicity in dogs]. Wei Sheng Yan Jiu. 2004;33(6):649–651. 15727166

[16] Emad A, Emad Y. Increased granulocyte-colony stimulating factor (G-CSF) and granulocyte-macrophage colony stimulating factor (GM-CSF) levels in BAL fluid from patients with sulfur mustard gas-induced pulmonary fibrosis. J Aerosol Med. 2007;20(3):352–360. doi: 10.1089/jam.2007.0590 17894541

[17] Amiri S, Ghazanfari T, Yaraee R, Serum levels of GM-CSF 20 years after sulfur mustard exposure: Sardasht-Iran Cohort Study. Int Immunopharmacol. 2009;9(13-14):1499–1503. doi: 10.1016/j.intimp.2009.08.023 19733693

[18] Chang YC, Soriano M, Hahn RA, Expression of cytokines and chemokines in mouse skin treated with sulfur mustard. Toxicol Appl Pharmacol. 2018;355:52–59. doi: 10.1016/j.taap.2018.06.008 29935281 PMC6438172

[19] Ryan JL, Heckler CE, Ling M, Curcumin for radiation dermatitis: a randomized, double-blind, placebo-controlled clinical trial of thirty breast cancer patients. Radiat Res. 2013;180(1):34–43. doi: 10.1667/RR3255.1 23745991 PMC3998827

[20] Shabeeb D, Musa AE, Abd Ali HS, Najafi M. Curcumin protects against radiotherapy-induced oxidative injury to the skin. Drug Des Devel Ther. 2020;14:3159–3163. doi: 10.2147/DDDT.S265228 PMC7429408 32848362

[21] Panahi Y, Sahebkar A, Parvin S, Saadat A. A randomized controlled trial on the anti-inflammatory effects of curcumin in patients with chronic sulphur mustard-induced cutaneous complications. Ann Clin Biochem. 2012;49(Pt 6):580–588. doi: 10.1258/acb.2012.012040 23038702

[22] Shiyovich A, Rosman Y, Krivoy A, [Long-term complications of sulfur mustard exposure: a therapeutic update]. Harefuah. 2014;153(3–4):199–205, 237. 24791566

[23] Demirel C, Kilciksiz S, Evirgen-Ayhan S, The preventive effect of N-acetylcysteine on radiation-induced dermatitis in a rat model. J BUON. 2010;15(3):577–582. 20941831

[24] Kim JA, Baker DG, Hahn SS, Topical use of N-acetylcysteine for reduction of skin reaction to radiation therapy. Semin Oncol. 1983;10(1 Suppl 1):86–92. 6403989

[25] Bobb AJ, Arfsten DP, Jederberg WW. N-acetyl-L-cysteine as prophylaxis against sulfur mustard. Mil Med. 2005;170(1):52–56. doi: 10.7205/milmed.170.1.52 15724855

[26] Sezigen S, Ivelik K, Ortatatli M, Victims of chemical terrorism, a family of four who were exposed to sulfur mustard. Toxicol Lett. 2019;303:9–15. doi: 10.1016/j.toxlet.2018.12.006 30572106

[27] Liang L, Hu D, Liu W, Celecoxib reduces skin damage after radiation: selective reduction of chemokine and receptor mRNA expression in irradiated skin but not in irradiated mammary tumor. Am J Clin Oncol. 2003;26(4):S114–S121. doi: 10.1097/01.coc.0000074149.95710.40 12902868

[28] Ghasemi A, Danesh B, Yazdani-Charati J, Hosseinimehr SJ. Randomized double-blind placebo-controlled trial of celecoxib for the prevention of skin toxicity in patients receiving radiation therapy for Breast cancer. Antiinflamm Antiallergy Agents Med Chem. 2018;17(1):57–67. doi: 10.2174/1871523017666180411162114 29651970

[29] Wormser U, Langenbach R, Peddada S, Reduced sulfur mustard-induced skin toxicity in cyclooxygenase-2 knockout and celecoxib-treated mice. Toxicol Appl Pharmacol. 2004;200(1):40–47. doi: 10.1016/j.taap.2004.03.013 15451306

[30] Wagner S, Lang S, Popp T, Evaluation of selective and non-selective cyclooxygenase inhibitors on sulfur mustard-induced pro-inflammatory cytokine formation in normal human epidermal keratinocytes. Toxicol Lett. 2019;312:109–117. doi: 10.1016/j.toxlet.2019.03.012 31048000

[31] Sawyer TW. N-Acetylcysteine as a treatment for sulphur mustard poisoning. Free Radic Biol Med. 2020;161:305–320. doi: 10.1016/j.freeradbiomed.2020.09.020 32980537 PMC7516373

[32] Han DW, Ji W, Lee JC, Efficacy of nebulized acetylcysteine for relieving symptoms and reducing usage of expectorants in patients with radiation pneumonitis. Thorac Cancer. 2019;10(2):243–248. doi: 10.1111/1759-7714.12938 30585684 PMC6360265

[33] Christofidou-Solomidou M, Pietrofesa RA, Arguiri E, Radiation mitigating properties of intranasally administered KL4 surfactant in a murine model of radiation-induced lung damage. Radiat Res. 2017;188(5):491–504. doi: 10.1667/rr14686.1 28877030 PMC5704933

[34] van Helden HP, Kuijpers WC, Diemel RV. Asthmalike symptoms following intratracheal exposure of guinea pigs to sulfur mustard aerosol: therapeutic efficacy of exogenous lung surfactant curosurf and salbutamol. Inhal Toxicol. 2004;16(8):537–548. doi: 10.1080/08958370490442520 15204745

[35] van Helden HP, van de Meent D, Oostdijk JP, Protection of rats against perfluoroisobutene (PFIB)-induced pulmonary edema by curosurf and N-acetylcysteine. Inhal Toxicol. 2004;16(8):549–564. doi: 10.1080/08958370490442575 15204746

[36] De Ruysscher D, Granton PV, Lieuwes NG, Nintedanib reduces radiation-induced microscopic lung fibrosis but this cannot be monitored by CT imaging: a preclinical study with a high precision image-guided irradiator. Radiother Oncol. 2017;124(3):482–487. doi: 10.1016/j.radonc.2017.07.014 28774597

[37] Qin W, Liu B, Yi M, Antifibrotic agent pirfenidone protects against development of radiation-induced pulmonary fibrosis in a murine model. Radiat Res. 2018;190(4):396–403. doi: 10.1667/RR15017.1 30016220

[38] Zamani N. Pirfenidone; can it be a new horizon for the treatment of pulmonary fibrosis in mustard gas-intoxicated patients? Daru. 2013;21(1):13. doi: 10.1186/2008-2231-21-13 23418997 PMC3599378

[39] Day RM, Davis TA, Barshishat-Kupper M, Enhanced hematopoietic protection from radiation by the combination of genistein and captopril. Int Immunopharmacol. 2013;15(2):348–356. doi: 10.1016/j.intimp.2012.12.029 23328620

[40] Sharma GP, Fish BL, Frei AC, Pharmacologic ACE-inhibition mitigates radiation-induced pneumonitis by suppressing ACE-expressing lung myeloid cells. Int J Radiat Oncol Biol Phys. 2022;113(1):177–191. doi: 10.1016/j.ijrobp.2022.01.023 35093482 PMC9018504

[41] Medhora M, Gao F, Jacobs ER, Moulder JE. Radiation damage to the lung: mitigation by angiotensin-converting enzyme (ACE) inhibitors. Respirology. 2012;17(1):66–71. doi: 10.1111/j.1440-1843.2011.02092.x 22023053 PMC3245332

[42] DiCarlo AL, Bandremer AC, Hollingsworth BA, Cutaneous radiation injuries: models, assessment and treatments. Radiat Res. 2020;194(3):315–344. doi: 10.1667/RADE-20-00120.1 32857831 PMC7525796

[43] Cassatt DR, Gorovets A, Karimi-Shah B, A trans-agency workshop on the pathophysiology of radiation-induced lung injury. Radiat Res. 2022;197(4):415–433. doi: 10.1667/RADE-21-00127.1 34342637 PMC9648426

[44] Weinberger B, Laskin JD, Sunil VR, Sulfur mustard-induced pulmonary injury: therapeutic approaches to mitigating toxicity. Pulm Pharmacol Ther. 2011;24(1):92–99. doi: 10.1016/j.pupt.2010.09.004 20851203 PMC3034290

[45] Pohanka M, Martinkova P, Brtnicky M, Kynicky J. Changes in the oxidative stress/anti-oxidant system after exposure to sulfur mustard and antioxidant strategies in the therapy, a review. Toxicol Mech Methods. 2017;27(6):408–416. doi: 10.1080/15376516.2017.1320695 28413899

[46] DiCarlo AL, Homer MJ, Coleman CN. United States medical preparedness for nuclear and radiological emergencies. J Radiol Prot. 2021;41(4):1–18. doi: 10.1088/1361-6498/ac0d3f PMC8648948 34153947

[47] Rios CI, Cassatt DR, Dicarlo AL, Building the strategic national stockpile through the NIAID Radiation Nuclear Countermeasures Program. Drug Dev Res. 2014;75(1):23–28. doi: 10.1002/ddr.21163 24648046 PMC8365557

[48] Singh VK, Romaine PL, Seed TM. Medical countermeasures for radiation exposure and related injuries: characterization of medicines, FDA-approval status and inclusion into the Strategic National Stockpile. Health Phys. 2015;108(6):607–630. doi: 10.1097/HP.0000000000000279 25905522 PMC4418776

[49] Food and Drug Administration (FDA), Center for Drug Evaluation and Research (CDER), Center for Biologics Evaluation and Research (CBER), Product Development Under the Animal Rule. Guidance for Industry; 2015.

[50] Chua HL, Plett PA, Sampson CH, Long-term hematopoietic stem cell damage in a murine model of the hematopoietic syndrome of the acute radiation syndrome. Health Phys. 2012;103(4):356–366. doi: 10.1097/HP.0b013e3182666d6f 22929468 PMC3743220

[51] Chua HL, Plett PA, Sampson CH, Survival efficacy of the PEGylated G-CSFs Maxy-G34 and neulasta in a mouse model of lethal H-ARS, and residual bone marrow damage in treated survivors. Health Phys. 2014;106(1):21–38. doi: 10.1097/HP.0b013e3182a4df10 24276547 PMC3843155

[52] Unthank JL, Miller SJ, Quickery AK, Delayed effects of acute radiation exposure in a murine model of the H-ARS: multiple-organ injury consequent to <10 Gy total body irradiation. Health Phys. 2015;109(5):511–521. doi: 10.1097/HP.0000000000000357 26425910 PMC4593322

[53] Chua HL, Plett PA, Fisher A, Lifelong residual bone marrow damage in murine survivors of the hematopoietic acute radiation syndrome (H-ARS): a compilation of studies comprising the Indiana University experience. Health Phys. 2019;116(4):546–557. doi: 10.1097/HP.0000000000000950 30789496 PMC6388630

[54] Unthank JL, Ortiz M, Trivedi H, Cardiac and renal delayed effects of acute radiation exposure: organ differences in vasculopathy, inflammation, senescence and oxidative balance. Radiat Res. 2019;191(5):383–397. doi: 10.1667/rr15130.1 30901530 PMC6538064

[55] Miller SJ, Chittajallu S, Sampson C, A potential role for excess tissue iron in development of cardiovascular delayed effects of acute radiation exposure. Health Phys. 2020;119(5):659–665. doi: 10.1097/HP.0000000000001314 32868705 PMC7541425

[56] Patterson AM, Plett PA, Chua HL, Development of a model of the acute and delayed effects of high dose radiation exposure in Jackson Diversity Outbred Mice; comparison to inbred C57BL/6 Mice. Health Phys. 2020;119(5):633–646. doi: 10.1097/HP.0000000000001344 32932286 PMC9374540

[57] Wu T, Plett PA, Chua HL, Immune reconstitution and thymic involution in the acute and delayed hematopoietic radiation syndromes. Health Phys. 2020;119(5):647–658. doi: 10.1097/HP.0000000000001352 32947490 PMC7541734

[58] Patterson AM, Sellamuthu R, Plett PA, Establishing pediatric mouse models of the hematopoietic acute radiation syndrome and the delayed effects of acute radiation exposure. Radiat Res. 2021;195(4):307–323. doi: 10.1667/RADE-20-00259.1 33577641

[59] Plett PA, Sampson CH, Chua HL, Establishing a murine model of the hematopoietic syndrome of the acute radiation syndrome. Health Phys. 2012;103(4):343–355. doi: 10.1097/HP.0b013e3182667309 22929467 PMC3743168

[60] Plett PA, Sampson CH, Chua HL, The H-ARS dose response relationship (DRR): validation and variables. Health Phys. 2015;109(5):391–398. doi: 10.1097/hp.0000000000000354 26425900 PMC4593318

[61] Garrett J, Sampson CH, Plett PA, Characterization and etiology of swollen muzzles in irradiated mice. Radiat Res. 2019;191(1):31–42. doi: 10.1667/rr14724.1 30339056

[62] Jones JW, Alloush J, Sellamuthu R, Effect of sex on biomarker response in a mouse model of the hematopoietic acute radiation syndrome. Health Phys. 2019;116(4):484–502. doi: 10.1097/hp.0000000000000961 30681425 PMC6384137

[63] Patterson AM, Vemula S, Plett PA, Age and sex divergence in hematopoietic radiosensitivity in aged mouse models of the hematopoietic acute radiation syndrome. Radiat Res. Published online July 14, 2022. doi: 10.1667/RADE-22-00071.1 PMC9512046 35834823

[64] Wirsdörfer F, Jendrossek V. Modeling DNA damage-induced pneumopathy in mice: insight from danger signaling cascades. Radiat Oncol. 2017;12(1):142. doi: 10.1186/s13014-017-0865-1 28836991 PMC5571607

[65] Perry MR, Neal M, Hawks R, A novel sulfur mustard (HD) vapor inhalation exposure model of pulmonary toxicity for the efficacy evaluation of candidate medical countermeasures. Inhal Toxicol. 2021;33(6-8):221–233. doi: 10.1080/08958378.2021.1951401 34396872 PMC8602763

[66] Beske PH, Wilhelm CM, Harvilchuck JA, A rodent model of sulfur mustard hematologic toxicity for the efficacy evaluation of candidate medical countermeasures. Mil Med. 2022;187(1-2):e106–e115.doi: 10.1093/milmed/usaa510 33346363 PMC8942104

[67] Burnett LR, Gabard AR, Robinson M, Biomolecular analysis of beta dose-dependent cutaneous radiation injury in a porcine model. Radiat Res. 2019;192(2):145–158. doi: 10.1667/rr14283.1 31166846

[68] Satyamitra M, Reyes Turcu FE, Pantoja-Galicia N, Wathen L. Challenges and strategies in the development of radiation biodosimetry tests for patient management. Radiat Res. 2021;196(5):455–467. doi: 10.1667/RADE-21-00072.1 34143223 PMC9923779

[69] Leiser OP, Hobbs EC, Sims AC, Beyond the list: bioagent-agnostic signatures could enable a more flexible and resilient biodefense posture than an approach based on priority agent lists alone. Pathogens. 2021;10(11):1497. doi: 10.3390/pathogens10111497 34832652 PMC8623450

[70] Sezigen S, Eyison RK, Ortatatli M, Myelosuppression and acute hematological complications of sulfur mustard exposure in victims of chemical terrorism. Toxicol Lett. 2020;318:92–98. doi: 10.1016/j.toxlet.2019.10.017 31678399

[71] Cai Y, Wang W, Liang H, Keratinocyte growth factor pretreatment prevents radiation-induced intestinal damage in a mouse model. Scand J Gastroenterol. 2013;48(4):419–426. doi: 10.3109/00365521.2013.772227 23464848

[72] Shea-Donohue T, Fasano A, Zhao A, An acute radiation syndrome (ARS) nonhuman primate (NHP) research platform: prolonged gastrointestinal (GI) dysfunction observed in NHPs surviving the acute heme and GI syndromes. Presented at the Fifty-fifth Annual Meeting of the Radiation Research Society; 2009.

[73] Farrell CL, Bready JV, Rex KL, Keratinocyte growth factor protects mice from chemotherapy and radiation-induced gastrointestinal injury and mortality. Cancer Res. 1998;58(5):933–939. 9500453

[74] Institute of Medicine. Drug repurposing and repositioning: workshop summary; The National Academies Collection: roundtable on translating genomic-based research for health; 2014. 24872991

